# Metabolic Biomarkers In Midtrimester Maternal Plasma Can Accurately Predict Adverse Pregnancy Outcome in Patients with SLE

**DOI:** 10.1038/s41598-019-51285-8

**Published:** 2019-10-23

**Authors:** Seung Mi Lee, Eun Mi Lee, Jin Kyun Park, Hae Sun Jeon, Sohee Oh, Subeen Hong, Young Mi Jung, Byoung Jae Kim, Sun Min Kim, Errol R. Norwitz, Eun Bong Lee, Souphaphone Louangsenlath, Chan-Wook Park, Jong Kwan Jun, Joong Shin Park, Do Yup Lee

**Affiliations:** 1 Department of Obstetrics and Gynecology, Seoul National University College of Medicine, Seoul, Korea; 2 Department of Agricultural Biotechnology, Center for Food and Bioconvergence, Research Institute for Agricultural and Life Sciences, Seoul National University, Seoul, Korea; 3 Division of Rheumatology, Department of Internal Medicine, Seoul National University College of Medicine, Seoul, Korea; 4 Department of Biostatistics, Seoul Metropolitan Government Seoul National University Boramae Medical Center, Seoul, Korea; 5 Department of Obstetrics and Gynecology, Seoul Metropolitan Government Seoul National University Boramae Medical Center, Seoul, Korea; 6 Department of Obstetrics and Gynecology, Tufts University School of Medicine, Boston, MA USA; 7 Department of Obstetrics and Gynecology, University of health science, Vientiane, Lao PDR

**Keywords:** Prognostic markers, Systemic lupus erythematosus

## Abstract

Patients with systemic lupus erythematosus (SLE) are at increased risk for adverse pregnancy outcome (APO). Accurate prediction of APO is critical to identify, counsel, and manage these high-risk patients. We undertook this study to identify novel biomarkers in mid-trimester maternal plasma to identify pregnant patients with SLE at increased risk of APOs. The study population consisted of pregnant women whose plasma was taken in mid-trimester and available for metabolic signature: (1) SLE and normal pregnancy outcome (Group 1, n = 21); (2) SLE with APO (Group 2, n = 12); and (3) healthy pregnant controls (Group 3, n = 10). Mid-trimester maternal plasma was analyzed for integrative profiles of primary metabolite and phospholipid using gas chromatography time-of-flight mass spectrometry (GC-TOF MS) and liquid chromatography Orbitrap mass spectrometry (LC-Orbitrap MS). For performance comparison and validation, plasma samples were analyzed for sFlt-1/PlGF ratio. In the study population, APO developed in 12 of 33 women with SLE (36%). Metabolite profiling of mid-trimester maternal plasma samples identified a total of 327 metabolites using GC-TOF MS and LC-Orbitrap MS. Partial least squares discriminant analysis (PLS-DA) showed clear discrimination among the profiles of SLE groups and healthy pregnant controls (Groups 1/2 vs. 3). Moreover, direct comparison between Groups 1 and 2 demonstrated that 4 primary metabolites and 13 lipid molecules were significantly different. Binary logistic regression analysis suggested a potential metabolic biomarker model that could discriminate Groups 1 and 2. Receiver operating characteristic (ROC) analysis revealed the best predictability for APO with the combination model of two metabolites (LysoPC C22:5 and tryptophan) with AUC of 0.944, comparable to the AUC of sFlt-1/PlGF (AUC 0.857). In conclusion, metabolic biomarkers in mid-trimester maternal plasma can accurately predict APO in patients with SLE.

## Introduction

Systemic lupus erythematosus (SLE) is a chronic multi-organ autoimmune disease, predominant in young women of childbearing age^1^. Since women with SLE usually have a normal fertility, SLE pregnancy is frequently encountered. Pregnant women with SLE can develop severe obstetric complications such as preterm birth, preeclampsia, or fetal loss^2^. Although maternal and obstetric outcomes have improved dramatically due to the advances in modern obstetrics^3^, adverse pregnancy outcomes (APOs) are relative common with a reported incidence of 19–57% even in recent studies^4,5^. Because of these obstetric complications, a pregnancy has been discouraged in SLE patients, at least when SLE is active. Nonetheless, it is critical to identify pregnant SLE patients at an increased risk of APOs to optimize their obstetric care. Previous studies have suggested that clinical factors such as the presence of lupus anticoagulant, use of antihypertensive medications, and higher disease activity are associated with APO^4,5^, but their diagnostic or prognostic value is limited^6,7^. Recent studies showed that the soluble fms-like tyrosine kinase 1 (sFlt-1)/placental growth factor (PlGF) imbalance is associated with APO in SLE patients^8,9^. However, its clinical implication to identify APO in SLE patients has not been fully elucidated.

A metabolome provides information on all biochemical activities, including substrate, product, and co-factors of all enzymatic reactions, in a given biological system at a single time point^10^. Changes in metabolome have been reported in patients with multiple sclerosis, SLE, and rheumatoid arthritis^11–16^. It is possible that metabolome of maternal blood of SLE patients might be influenced by the developing mechanisms of APO and therefore a subtle change in the maternal metabolome may help to identify those SLE patients at a high risk of APOs.

In the current study, an integrative metabolic profiling was conducted to acquire comprehensive information on primary metabolites and lipids. Primary metabolites are the constituents of central carbon/nitrogen metabolism. This includes amino acid and carbohydrates (the intermediates of glycolysis) that play an essential role in bioenergetics and the biosynthesis of molecular building block. Lipid molecules including phospholipids (PLs) are essential components of biological membranes and moreover precursors of numerous signaling molecules. The disturbed lipid metabolism has been reported in autoimmune disorders such as Guillain–Barré syndrome (GBS), multiple sclerosis (MS)^17^, and rheumatoid arthritis (RA)^18^.

The objectives of this study were: (1) to explore the distinctive metabolic physiology in pregnant patients with SLE compared to healthy pregnant controls, (2) to identify discriminatory biomarkers to predict APOs in patients with SLE, and (3) to assess the association between metabolic profiling and symptom severity of APOs using mid-trimester maternal plasma of SLE women.

## Results

### Study population

A total of 33 pregnant SLE patients were included. APO developed in 12 (36%) of SLE patients. SLE patients were grouped into SLE patients with normal pregnancy outcome (Group 1, n = 21) and SLE patients with APO (Group 2, n = 12). The control (Group 3) included 10 matched healthy pregnancies (Group 3). The median maternal age, gestational age at sampling, and BMI at sampling were not different among the three groups. SLE women with APO (Group 2) had a higher frequency of antiphospholipid antibody syndrome and hypertension than those with normal pregnancy outcome (Group 1). Group 2 had lower gestational age at delivery and birthweight than those in Groups 1 (Table 1).

**Table 1:** Characteristics of the study population.

	Group 1 SLE with normal pregnancy outcome (n = 21)	Group 2 SLE with adverse pregnancy outcome (n = 12)	Group 3 Healthy pregnant controls (n = 10)	P^a^
**Baseline characteristics**				
Maternal age (y)*	34 (27–40)	33 (28–38)	35 (28–38)	NS
Nulliparity	12 (57%)	8 (67%)	2 (20%)	NS
Pregnancy after assisted reproduction	1 (5%)	0 (0%)	0 (0%)	NS
Gestational age at sampling^b^	18.3 (15.7–22.6)	18.1 (16.3–22.0)	18.0 (17.0–22.7)	NS
BMI at sampling^b^	23.4 (18.3–37.8)	23.1 (17.4–30.8)	22.8 (21.0–28.3)	NS
Lupus nephritis	10 (48%)	5 (42%)		NS
Antiphospholipid antibody syndrome^c^	1 (5%)	4 (33%)		<0.05
Hypertension at sampling ^b,d^	2 (10%)	7 (58%)		<0.01
**Medication at sampling**				
Hydroxychloroquine	5 (24%)	3 (25%)		NS
Sulfasalazine	1 (5%)	0 (0%)		NS
Aspirin	0 (0%)	4 (33%)		<0.05
Heparin	0 (0%)	4 (33%)		<0.05
Prednisolone	14 (67%)	10 (83%)		NS
Presence of lupus anticoagulant^e^	2/17 (12%)	4 (33%)		NS
WBC counts < 3,000/mm^3 e^	0 (0%)	0 (0%)		(—)
Platelet counts < 100,000/mm^3 e^	0 (0%)	4 (33%)		<0.05
C3 (mg/dL)^e^	118 (88–171) (n = 19)	94 (32–147) (n = 11)		NS
C4 (mg/dL)^e^	20 (10–33) (n = 19)	11.5 (7–47) (n = 11)		<0.01
Anti ds-DNA (IU/mL)^e^	6.6 (<1.0–58.6) (n = 20)	9.8 (<1.0–174) (n = 11)		NS
**Pregnancy outcomes**				
Gestational age at delivery (wk)*	38.6 (31.7–40.7)	29.6 (20.6–40.3)	38.8 (38.0–40.7)	<0.005
Birthweight (g)*	2990 (1650–4040)	1130 (40–2590)	3445 (2860–3690)	<0.001
Cesarean delivery	9 (43%)	6 (50%)	5 (50%)	NS
Sex (male)	13 (62%)	4 (33%)	3 (30%)	NS
SLE flare during pregnancy^f^	2 (10%)	6 (50%)		<0.05

*data are presented as median (range); NS, not significant; wk, weeks.

P^a^, comparison between Groups 1 and 2.

^b^at sampling: at mid-trimester maternal blood sampling.

^c^Diagnosis made by attending physician (Rheumatologist) before mid-trimester maternal blood sampling.

^d^High blood pressure: history of hypertension or taking anti-hypertensive medication at mid-trimester maternal blood sampling.

^e^Result before mid-trimester maternal blood sampling or within one year after delivery.

^f^Based on review of medical records incorporating new or worsening clinical manifestations, laboratory measures, and medication doses or addition of new medications.

The most common APO was preterm delivery before 36 weeks (67%), followed by preeclampsia (58%), small for gestational age at birth (50%) and intrauterine fetal death (25%). A neonatal death before discharge was not observed. Of the 12 APOs, 7 (58%) were classified as severe APOs (Table 2).

**Table 2:** Development of adverse pregnancy outcome.

	Group 2 SLE with adverse pregnancy outcome (n = 12)
**Severe adverse pregnancy outcome**	
Fetal death in utero	3 (25%)
Preeclampsia requiring delivery <34 weeks	2 (17%)
Neonatal death before discharge	0 (0%)
Indicated preterm delivery (<30weeks)	6 (50%)
**Moderate adverse pregnancy outcome**	
Preeclampsia resulting in delivery ≥34 weeks	5 (42%)
Indicated preterm delivery (30–36weeks)	2 (17%)
Small for gestational age at birth (<5^th^)	6 (50%)

### Higher angiogenic factors in patients with APO

**Figure 1:**
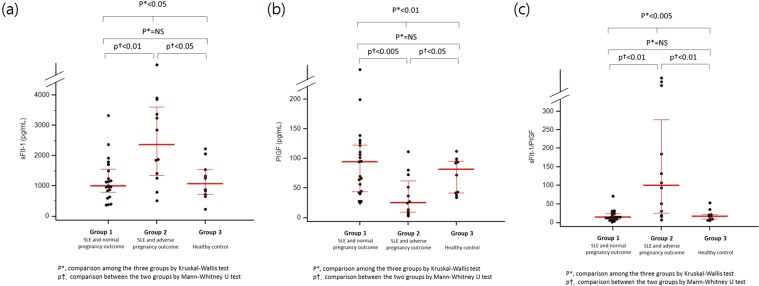
The concentrations of sFlt-1 and PlGF and sFlt-1/PlGF ratio in the study population. **(a)** sFlt-1. **(b)** PlGF. **(c)** The sFlt-1/PlGF ratio.

The median concentration of sFlt-1 and the sFlt-1/PlGF ratio in mid-trimester maternal plasma was significantly higher in women in Group 2 compared with Group 1 or Group 3 (Fig. 1). While SLE with normal pregnancy outcome did not differ from the healthy pregnant controls, SLE with APO had significantly higher levels of sFlt1.

### Distinctive metabolome of mid-trimester maternal plasma of SLE patients

**Figure 2:**
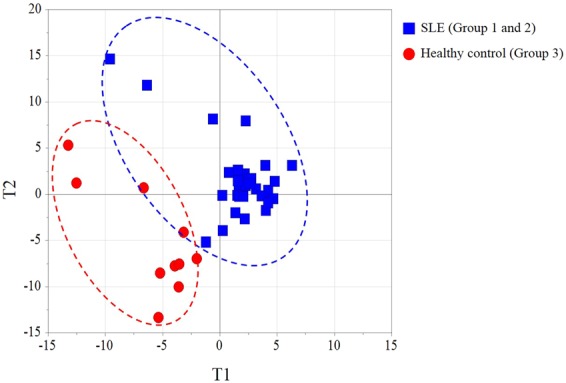
Multivariate statistical model by partial least squares discriminant analysis (PLS-DA) analysis showing distinctive plasma metabolite profiles between the SLE groups and healthy pregnant controls. T1 and T2 indicates are the vectors, which explain the two largest degree of variation in the model. R2Y (0.991) is cumulative goodness-of-fit and Q2 proposes model predictability (0.725).

Of 105 primary metabolites, 6 (5.7%) compounds were present at significantly different levels in the SLE groups as compared to healthy pregnant controls, namely 2-deoxytetronic acid, erythritol, pyruvic acid, uric acid, valine, xylose (Mann-Whitney U test, p value < 0.05). Likewise, lipidomic dysregulation in the SLE groups was evidenced in which 26 (11.7%) of lipid molecules were significantly altered compared to healthy pregnant controls. PLS-DA model with seven-fold cross validation showed high level of an explained variance (R2Y = 0.991) and predictability (Q2 = 0.725). The resultant score scatter plot presented distinctive clusters between SLE groups and healthy pregnant controls (Fig. 2).

### Unique metabolic signatures of APO

Next, we examined if metabolite profiles were unique to APO among the SLE patients. Direct comparison between Groups 1 and 2 demonstrated that 55 (16.8%) of metabolites were significantly different (Supplementary Table 1). For correction of multiple comparisons, false discovery rate (FDR) was estimated and consequent criteria (q-value < 0.05) were applied for the comparison of metabolites. A total of 4 primary metabolites and 13 lipid molecules still remained significantly different between Group 1 and 2 (Supplementary Table 1).

Binary logistic regression analysis was used to identify potential metabolic biomarkers present in the mid-trimester maternal plasma that could predict a subsequent APO in patients with SLE. A total of 17 metabolites that were at different levels between Groups 1 and 2 (FDR, q-value < 0.05) in the univariate analysis were entered into a multivariate logistic regression analysis. The analysis with forward selection was represented by risk of APO = 5.55 + (1816.18 * LysoPC C22:5) + (−416.57 * Tryptophan). The ROC curve analysis presenting the combined panel of the metabolic markers showed excellent performance (AUC: 0.944, 95% confidence interval [CI]: 0.805–0.994).

**Figure 3:**
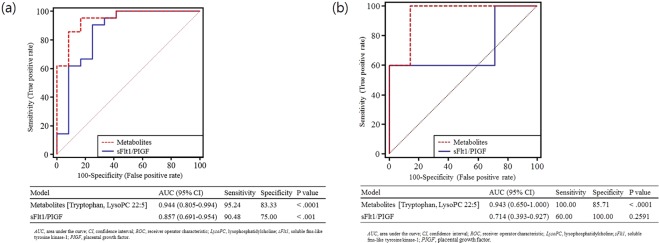
Receiver operating characteristic (ROC) curves of plasma metabolic biomarkers (the binary logistic regression function of tryptophan and lysoPC 22:5) and sFlt-1/PlGF to predict adverse pregnancy outcome in women with SLE. Optimal cutoff is determined based on the closest to top-left corner. **(a)** ROC curves to predict adverse pregnancy outcome in women with SLE (discrimination between Groups 1 and 2). **(b)** ROC curves to predict severe adverse pregnancy outcome in women with SLE and adverse pregnancy outcome (discrimination between moderate and severe adverse pregnancy outcome).

For validation of the algorithm, approximately 50% of the dataset were randomly selected and examined. This step was repeated three times and the result confirmed a robust performance for predicting APO among SLE patients (AUCs: 0.96, 0.99, and 0.97). Moreover, pair-wise comparison of the ROC curves showed that the metabolic signature had a higher AUC than the one of the sFlt1/PIGF ratio although the comparison was not statistically significant (*p* = 0.209) (Fig. 3(a)). Considering potential effects of confounders on the discrimination, the predictive efficacies of the two models were evaluated after adjustment of confounding variables. Indeed, the metabolic biomarker remained significant whereas that of sFlt-1/PlGF failed to reach statistical criteria (Table 3).

**Table 3:** Univariate and multivariate analyses predicting adverse pregnancy outcome in pregnant women with SLE.

Characteristics	(1) Metabolites (Tryptophan and LysoPC 22:5)	*P*
Unadjusted	2.719 (1.315–5.626)	<0.01
Model 1	2.614 (1.247–5.483)	<0.05
Model 2	2.410 (1.183–4.907)	<0.05
Model 3	2.239 (1.095–4.578)	<0.05
Model 4	2.132 (1.038–4.377)	<0.05
	(2) sFlt-1/PlGF	
Unadjusted	1.045 (1.005–1.087)	<0.05
Model 1	1.034 (0.994–1.075)	0.098
Model 2	1.030 (0.987–1.075)	0.176
Model 3	1.021 (0.973–1.072)	0.394
Model 4	1.016 (0.987–1.046)	0.286

SLE, systemic lupus erythematosus; LysoPC, lysophosphatidylcholine; sFlt1, soluble fms-like tyrosine kinase-1; PIGF, placental growth factor.

Model 1: adjusted for hypertension at sampling.

Model 2: adjusted for hypertension at sampling and antiphospholipid antibody syndrome.

Model 3: adjusted for hypertension at sampling, antiphospholipid antibody syndrome, and use of heparin/aspirin at sampling.

Model 4: adjusted for hypertension at sampling, antiphospholipid antibody syndrome, use of heparin/aspirin at sampling, platelet counts, and the level of C4.

### Severity-dependent metabolomic dynamics in SLE patients with subsequent APO

Further interrogation was done on compositional differences of individual metabolites between moderate APO and severe APO in SLE patients in Group 2. A total of 30 primary metabolites and lipid molecules were present at significantly different levels between two groups (Mann-Whitney U test, p value < 0.05). The SLE patients with severe APO were best characterized by reduced amino acids (e.g. methionine, tryptophan, and valine). In contrast, there was an increase in lipophilic compounds including saturated fatty acid (palmitic acid), polyunsaturated fatty acids (oleic acid, linoleic acid and arachidonic acid), and branched fatty acid esters of hydroxy fatty acids (FAHFAs). Among them, tryptophan, a biomarker constituent, showed significantly lower level even after multiple comparison correction (FDR, q-value = <0.001).

**Figure 4:**
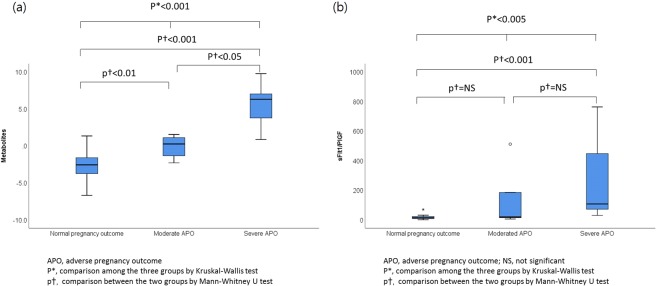
The plasma metabolic biomarkers (the binary logistic regression function of tryptophan and lysoPC 22:5) and sFlt-1/PlGF according to pregnancy outcomes in women with SLE. (**a)** The plasma metabolic biomarkers. **(b)** The sFlt-1/PlGF ratio.

We then examined whether the binary logistic regression model, which was applied to the discrimination model between Groups 1 and 2, could also discriminate the SLE patients according to the severity of APO. The AUC value of the combination model consisting of the two metabolites (Tryptophan and LysoPC C22:5) was 0.943 (95% CI, 0.650–1.000, p < 0.001) with sensitivity (100%) and specificity (85.7%), which was also superior to the predictability based on the sFlt1/PIGF ratio (AUC: 0.714, 95% CI: 0.393–0.927) (Fig. 3(b)). When plotted against pregnancy outcomes, the combination model consisting of the two metabolites and sFlt1/PIGF ratio increased according to the severity of APO (Fig. 4, p < 0.001 for the combination model consisting of the two metabolites; p < 0.005 for sFlt1/PIGF ratio by Kruskal-Wallis test).

Among the pregnant women with SLE, 24% (8/33) experienced SLE flare during pregnancy (Table 1). SLE patients with APO also experienced SLE flare more frequently than SLE patients with normal pregnancy outcome. In addition, the two metabolites and sFlt-1/PlGF ratio were different between who experienced SLE flare and those who did not. The AUC of the combination model consisting of the two metabolites and sFlt-1/PlGF ratio for SLE flare were 0.882 and 0.863, respectively (p < 0.001 for both).

## Discussion

The principal findings of this study were: **(**1) 12 (36%) of 33 SLE women developed APO; (2) Metabolite profiling of mid-trimester maternal plasma showed distinctive and discriminatory metabolic physiology between healthy pregnant controls and SLE patients; (3) Biomarker panels using a combination of two biomarkers (Tryptophan and LysoPC C22:5) in mid-trimester maternal plasma accurately predicted an APO among pregnant women with SLE; and (4) The metabolomic biomarkers were closely associated with the severity of APO in patients with SLE and APO.

Obstetric outcomes in pregnant women with SLE have improved dramatically in recent decades. In this study, APO was still present in 36% of SLE pregnancy, consistent with the previously presorted prevalence of 20–50%^4,5,19^. For clinicians, it is important to accurately identify, counsel, and manage these patients in order to optimize their pregnancy outcome. Multiple studies suggested that renal involvement, SLE disease activity and the presence of antiphospholipid antibodies are associated with APO during SLE pregnancy^4,5^. However, their clinical laboratory parameters are rather “static or fixed” and do not reflect the real time dynamic physiology changes during SLE pregnancy.

The current study focused, therefore, on identifying a distinctive metabolome as novel biomarkers in SLE pregnancy. It was noted that the majority of dysregulated metabolites were lipid molecules (26 lipids of 32 molecules that achieved statistical significance). Among these, PtdIns (C40:5) showed the highest fold change (2.9-fold increase in the SLE group). Phosphatidylinositol (PtdIns) is the most abundant phosphoinositides in mammalian cells, and its dysregulation has been implicated in a number of human diseases^20^. In particular, dysregulation in PtdIns signaling and *de novo* synthesis has been reported to induce endoplasmic reticulum (ER) stress^21^. The occurrence of ER stress has been linked to autoimmune diseases, including SLE, where it may play a role in affecting the course and outcome of this disorder^22^.

Thereafter, we investigated the lipid profiles in SLE patients with APO, which were best characterized by a significant upregulation in acylcarnitines and lysophospholipids with relatively long acyl-chains. Acylcarnitine is a mediator for fatty acids translocation between the cytosol and mitochondria, which allows β-oxidation for energy production through the TCA cycle and oxidative phosphorylation^23^. Aberrant levels of acylcarnitines can lead to dysregulation of one of several carnitine acyl-CoA transferases, which causes disturbed fatty acid oxidation processing^24^. Abnormal β-oxidation may interfere with the TCA cycle leading to insulin resistance^23^, which has been correlated with adverse clinical events in patients with SLE^25^. A previous metabolomic study also reported potential disturbances in β-oxidation in the SLE group (non-pregnant patients) compared to healthy controls^15^. The atypical regulation in fatty acid metabolism was further evidenced by the significant increases in a range of fatty acids. In contrast, in this study, levels of various types of amino acids (including lysine, methionine, proline, tryptophan and tyrosine) were significantly lower in SLE patients with APO. This is consistent with the previous reports suggesting an impairment of energy metabolism in patients with SLE (not including pregnant patients)^15^. Taken together, these data suggest that dysregulation in β-oxidation and consequent abnormalities in energy metabolism are not only a general metabolic feature of SLE, but may be associated more specifically with APO in pregnant patients with SLE. To some extent, the metabolic dysregulation in SLE was comparable with a previous study that proposed the metabolic marker for the development of preeclampsia in non-SLE pregnant women^26^. For instance, glycerol, the most significantly altered metabolite in the preeclampsia case study, showed moderate level of decrease in SLE compared to controls in our study. Besides, the significant alteration in pyruvate and valine was identical in both studies.

We then identified a biomarker panel in pregnant patients with SLE that can accurately discriminate APO from normal outcome (Group 1 vs 2). Binary logistic regression analysis with forward selection method prioritized LysoPC (C22:5) and the aromatic amino acid, tryptophan, both of which showed APO-specific metabolic features. The linear combination of these two metabolic markers achieved the best predictability with 95% sensitivity and 83% specificity for the prediction of APO, which was superior to the sFlt1/PIGF ratio.

Defective trophoblast invasion and remodeling of the maternal spiral arteries appears to be the pathologic hallmark of early preeclampsia^27^. In this model, uteroplacental insufficiency stimulates placental production of anti-angiogenic factors, including sFlt-1, which subsequently sequesters PlGF^28,29^. In agreement with this pathophysiology, sFlt-1 and PlGF appear to be the most useful biomarkers for the prediction of preeclampsia in high-risk pregnant women. Recently, Kim *et al*. reported that circulating angiogenic factors measured early in pregnancy can rule out the development of severe APO among patient with SLE and/or antiphospholipid antibody syndrome. In this cohort, the combination of sFlt-1 and PlGF measured at 16–19 weeks was most predictive of severe APO^9^. In the current study, we identified metabolic biomarkers in mid-trimester maternal plasma that can accurately predict APO in pregnant women with SLE. Our combination model consisting of two metabolites showed predictability comparable to that of sFlt-1/PlGF. To our knowledge, this is the first report to describe metabolic biomarkers in the mid-trimester of pregnancy to predict APO in women with SLE.

Interestingly, the identical model was also able to discriminate the severity of the APO among patients with SLE (moderate vs severe APO) with 100% sensitivity and 85.7% specificity. Likewise, the model outperformed the ability of the sFlt1/PIGF ratio to predict APO severity in patients with SLE. Given their known biochemistry, both of these two metabolic biomarkers (Tryptophan and LysoPC C22:5) are biologically plausible causative agents of disease severity. LysoPC is a product of partial hydrolysis of PC typically by phospholipase A2. Among its known pathologically relevancies are the activation of endothelial cells^30,31^ and stimulation of phagocyte recruitment^32^, both of which have been associated with the pathogenesis of SLE in the context of immune dysregulation^33–35^. In our study, LysoPCs with long acyl-chains were significantly upregulated in SLE patients with APO, including LysoPCs (C20:4, C20:5, C22:5, and C22:6). Of note, LysoPC with acyl-chain of docosahexaenoic acid (C22:5), a component of the biomarker panel, has also been identified as a marker of ageing^36^ and hepatocarcinogenesis^37^. Earlier studies reported that enhanced tryptophan degradation in SLE patients is associated with functional impairment of T cell^38^, and a more recent study proposed that kynurenine (a metabolite of tryptophan) is altered in patients with SLE who have severe fatigue^39^.

This study has several limitations. First, given the small number of cases, we could not perform comprehensive validation studies. Further studies in larger population are needed to verify the clinical utility of these biomarkers. Second, we have shown that the predictability of lysoPC C22:5 and tryptophan was comparable to that of sFlt-1/PlGF. Whether the addition of lysoPC C22:5 and tryptophan to sFlt-1/PlGF may enhance the performance of the prediction model even further is not known. Third, there remains no proven method to effectively prevent APO in pregnant women with SLE. Low-dose aspirin may reduce the risk of preeclampsia in patients at high risk^40,41^, and women with antiphospholipid antibody syndrome are known to have better pregnancy outcomes if on low-dose aspirin and heparin prophylaxis^42^. Whether this is true also of women with SLE is not known. Lastly, we evaluated mid-trimester maternal plasma (15–24 weeks) for prediction of APO. The predictive power for APO has been different among gestational age according to various biomarkers. In the study Levine *et al*., the soluble endoglin levels were significantly higher beginning at 17–20 weeks of gestation in women with subsequent preterm preeclampsia, whereas the endoglin levels were higher beginning at 25–28 weeks of gestation in women with subsequent term preeclampsia^43^. In the study of biomarkers in SLE, the angiogenic factors (sFlt-1 and PlGF) began to rise as early as 1st trimester^9^. Based on these evidences, the current study targeted mid-trimester maternal plasma for the exploration of various metabolites. The predictive power of plasma metabolites in early pregnancy also needs to be evaluated in further studies.

In conclusion, we have identified a metabolomic profile that can accurately predict the development of APO and the severity of APO in pregnant patients with SLE.

## Methods

### Study design

The study population were derived from the perinatal database in Seoul National University Hospital which enrolls pregnant women during antenatal care, and 33 pregnant women with SLE who met the following criteria were included: (1) singleton pregnancy; (2) mid-trimester (15–24 weeks) maternal plasma was available for metabolic profiling; and (3) followed up through delivery at Seoul National University Hospital. As controls, 10 normal pregnant women who were matched for gestational age at sample collection and for maternal age were selected from the perinatal database at a ratio 3:1. The study population was divided into 3 groups as follows: (1) SLE patients with normal pregnancy outcome (Group 1); (2) SLE patients with APO (Group 2); and (3) healthy pregnant controls (Group 3). Stored mid-trimester maternal plasma samples were analyzed for a metabolic profiling. The institutional review board of Seoul National University Hospital Clinical Research Institute approved this study (IRB No. 1611–126–812) and patients provided their written informed consent for the collection and use of clinical information and blood samples for research purpose. All methods were performed in accordance with the relevant guidelines and regulations.

### Definition of APO

APO was defined by the criteria of PROMISSE study with minor modification^9^, and included one or more of the following: (1) intrauterine fetal death unexplained by chromosomal abnormality, major malformation, or congenital infection; (2) preeclampsia; (3) neonatal death before discharge from hospital; (4) indicated preterm delivery before 36 weeks of gestation; and/or (5) small for gestational age at birth (birth weight <5^th^ percentile). APO was further classified into moderate or severe APO, as outlines in the PROMISSE study^9^. Severe APO was defined as the presence of at least one of the following: (1) intrauterine fetal death; (2) preeclampsia requiring delivery before 34 weeks of gestation; (3) neonatal death before discharge from hospital; and/or (4) indicated preterm birth less than 30 weeks of gestation. Moderate APO was defined as: (1) preeclampsia resulting in delivery ≥34 weeks of gestation, (2) indicated preterm birth between 30 and 36 weeks of gestation, and/or (3) small for gestational age at birth (birth weight <5^th^ percentile). Adverse outcomes were determined by review of the medical record.

### Metabolic profiling of mid-trimester maternal plasma

The mid-trimester maternal plasma was taken and centrifuged, and the supernatants were stored at −70 °C until assayed. The stored mid-trimester maternal plasma was analyzed for integrative profiles of 105 primary metabolites and 222 lipid molecules using gas chromatography time-of-flight mass spectrometry (GC-TOF MS) and liquid chromatography Orbitrap mass spectrometry (LC-Orbitrap MS). [See Supplementary File for further details of methodology].

Soluble fms-like tyrosine kinase-1 (sFlt-1)/Placental growth factor (PlGF) were measured with high-sensitive multiplex array (Meso Scale Discovery (MSD) V-PLEX Angiogenesis Panel 1 Human kit, MSD, USA), according to the manufacturer’ instruction.

### Statistical analysis

Proportions were compared with Fisher’s exact test, and comparisons of continuous variables between groups were performed with a Mann-Whitney U test or Kruskall-Wallis test as appropriate. A p-value < 0.05 was considered significant.

Metabolomic data was normalized based on MSTUS method^44^ implemented in NOREVA (http://idrb.zju.edu.cn/noreva/)^45^ prior to statistical analysis. Univariate statistical analysis was conducted based on Mann-Whitney U test using IMB SPSS Statistics for Windows, version 25.0 (IBM Corp., Armonk, N.Y., USA). For correction for multiple testing, significance analysis of microarray (SAM) was applied to control false discovery rate (FDR). Multivariate statistical analysis was performed for partial least squares-discriminant analysis (PLS-DA) by SIMCA 15 (Umetrics AB, Umea, Sweden). Binary logistic regression analysis with forward selection was conducted using IMB SPSS Statistics for Windows, version 25.0 (IBM Corp., Armonk, N.Y., USA). Receiver operating characteristic (ROC) analysis was performed using MedCalc for Windows, version 12.7.0.0 (MedCalc Software, Ostend, Belgium).

## Supplementary information

supplementary method

supplementary tables
