# Supplementary material for: Metabolic Biomarkers In Midtrimester Maternal Plasma Can Accurately Predict Adverse Pregnancy Outcome in Patients with SLE
Source: Sci Rep. 2019 Oct 23;9:15169. doi: 10.1038/s41598-019-51285-8 (PMC6811572; doi:10.1038/s41598-019-51285-8)
Supplement: Supplementary file 1 — supplementary method [file 41598_2019_51285_MOESM1_ESM.pdf]

Metabolic Biomarkers In Midtrimester Maternal Plasma Can Accurately Predict Adverse Pregnancy Outcome in Patients with SLE

Seung Mi Lee, MD, PhD[1], Eun Mi Lee, Ms[2], Jin Kyun Park, MD, PhD [3], Hae Sun Jeon, MD[1], Sohee Oh, PhD[4], Subeen Hong, MD[1], Young Mi Jung, MD[1], Byoung Jae Kim, MD[1,5], Sun Min Kim, MD, PhD[1,5], Errol R. Norwitz, MD, PhD[6], Eun Bong Lee, MD, PhD [3], Souphaphone Louangsenlath, MD[7], Chan-Wook Park, MD, PhD [1], Jong Kwan Jun, MD, PhD [1], Joong Shin Park, MD, PhD[1*], Do Yup Lee, PhD[2*]

[1] Department of Obstetrics and Gynecology, Seoul National University College of Medicine, Seoul, Korea

[2] Department of Agricultural Biotechnology, Seoul National University, Seoul, Korea

[3] Division of Rheumatology, Department of Internal Medicine, Seoul National University College of Medicine, Seoul, Korea

[4] Department of Biostatistics, Seoul Metropolitan Government Seoul National University Boramae Medical Center, Seoul, Korea

[5] Department of Obstetrics and Gynecology, Seoul Metropolitan Government Seoul National University Boramae Medical Center, Seoul, Korea

[6] Department of Obstetrics and Gynecology, Tufts University School of Medicine, Boston, MA, U.S.A

[7] Department of Obstetrics and Gynecology, University of health science, Lao PDR

Supplementary Method

Metabolite extraction Analytical processes were performed in random order, including extraction, derivatization, reconstitution, and MS analysis. Metabolite was extracted by mixing 50 μL of mid-trimester maternal serum and tertiary solvent mixture (750 μL, methanol:isopropanol:water, 3:3:2, v/v/v) followed by 10-min sonication and centrifugation (13,200 rpm for 5 minutes at 4°C). Aliquot (750 μL) was transferred into a new 1.5-mL tube and concentrated to complete dryness in a speed vacuum concentrator (SCANVAC, Korea). The dried extracts were stored at -80°C until GC-TOF MS analysis.

For lipid analysis, the Folch method was applied with minor modification as follows: A 50 μL of plasma sample was vigorously mixed with 225 μL of MeOH for 10 s followed by the addition of chloroform (450 μL) and incubation for 1 hr. Water (187.5 μL). After phase separation, lower phase was transferred to a new vial and concentrated to complete dryness (SCANVAC, Korea). Dried extract was stored at -80°C until reconstitution for LC-Orbitrap MS analysis.

GC-TOF analysis for primary metabolite profiling The first derivatization process was done with 5 μL of 40 mg/mL methoxyamine hydrochloride (Sigma-Aldrich, St. Louis, MO, USA) in pyridine (Thermo, USA) (90 min at 200 rpm at 37°C). The

mixture was derivatized with 45 μL of N-methyl-N-trimethylsilyltrifluoroacetamide (MSTFA + 1% TMCS; Thermo, USA) for 1 h at 200 rpm at 30°C [1-3]. Internal retention index markers (13 fatty acid methyl esters) was added to the derivatization mixture. Following the derivatization, 0.5 μL of sample was injected using an Agilent 7693 ALS (Agilent Technologies, Wilmington, DE, USA). Metabolites were chromatographically separated by an Agilent 7890B gas chromatography (Agilent Technologies) with an RTX-5Sil MS column (Restek, Gellefonte, PA, USA). Mass spectrometry analysis was conducted on a Leco Pegasus HT time of flight mass spectrometer controlled by Chroma TOF software 4.50 version (LECO, St. Joseph, MI, USA). The GC oven temperatures were programmed as follows: held at 50°C for 1min, ramped to 330°C at 20°C/min, and held constant for 5 min. Acquired data were further processed based on *Binbase database* [3], which generate chemical name, spectra information, retention time, unique ion, and peak height in excel format. A mixture of 33 pure standard compounds was analyzed every 10 samples for quality control purpose.

LC-Orbitrap MS analysis The dried extract was reconstituted with 50 μl of acetonitrile (70%) for LC-Orbitrap MS analysis. The lipid was chromatographically separated with a 150 mm × 2.1 mm UPLC BEH 1.7-μm C18 column (Waters Corporation, Milford, MA, USA) equipped with 5.0 mm × 2.1 mm UPLC BEH 1.7 μm

C18 VanGuard Pre-Column (Waters Corporation, Milford, MA, USA). The liquid chromatography was conducted by Ultimate-3000 UPLC system (Thermo Fisher Scientific Inc., Waltham, MA, USA).

The lipid extract was chromatographically separated by binary solvent system consisting of solvent A (water with 10mM ammonium formate and 0.2% formic acid) and solvent B (acetonitrile with 0.2% formic acid). The gradient was as follows: equilibration in 10% solvent B for 1 min, 10–75% solvent B gradient until 7.5 min, 75–95% solvent B gradient until 7.6 min, 95% solvent B until 10.4 min, 95-10% solvent B gradient until 10.5min, and re-equilibration in 10% solvent B until 16 min.

Ten microliter of the lipid extract was used for both MS1 and MS/MS analysis. MS analysis was executed with positive and negative ionization mode using Q-Exactive Plus Orbitrap (Thermo Fisher Scientific Inc., Waltham, MA, USA). MS/MS analysis was conducted in data-dependent manner (HCD, 30 eV). Raw data (.raw format) were transformed to Analysis Base File (ABF) format by Reifycs Abf Converter (http://www.reifycs.com/AbfConverter/index.html). Data process (peak detection, smoothing, and deconvolution) and compound identification were done by the MS-DIAL software and Lipid Blast library (MS1 tolerance: 0.005 Da; MS2, 0.0075 Da; similarity score, 70%) [4, 5]. A total of 222 lipids were identified and used for subsequent

statistical analysis following manual inspection on peak quality by Tracer Finder (Thermo Fisher Scientific Inc., Waltham, MA, USA).

Refernces

1. Lee DY, Fiehn O. High quality metabolomic data for Chlamydomonas reinhardtii. Plant methods 2008;4:7.
2. Lee J-E, Cho YU, Kim KH, Lee DY. Distinctive metabolomic responses of Chlamydomonas reinhardtii to the chemical elicitation by methyl jasmonate and salicylic acid. Process Biochemistry 2016;51:1147-54.
3. Fiehn O, Wohlgemuth G, Scholz M. Setup and annotation of metabolomic experiments by integrating biological and mass spectrometric metadata. International Workshop on Data Integration in the Life Sciences: Springer, 2005.
4. Tsugawa H, Cajka T, Kind T, et al. MS-DIAL: data-independent MS/MS deconvolution for comprehensive metabolome analysis. Nat Methods 2015;12:523-6.
5. Kind T, Liu K-H, Lee DY, DeFelice B, Meissen JK, Fiehn O. LipidBlast in silico tandem mass spectrometry database for lipid identification. Nature Methods 2013;10:755-58.
6. Dalby AR, Karpievitch YV, Nikolic SB, Wilson R, Sharman JE, Edwards LM. Metabolomics Data Normalization with EigenMS. PLoS ONE 2014;9:e116221.
7. Li B, Tang J, Yang Q, et al. NOREVA: normalization and evaluation of MS-based metabolomics data. Nucleic Acids Research 2017;45:W162-W70.
8. Saeed A. TM4: a free, open-source system for microarray data management and analysis. BioTechniques 2003;34:374-78.
