# Supplementary material for: Metabolic Biomarkers In Midtrimester Maternal Plasma Can Accurately Predict Adverse Pregnancy Outcome in Patients with SLE
Source: Sci Rep. 2019 Oct 23;9:15169. doi: 10.1038/s41598-019-51285-8 (PMC6811572; doi:10.1038/s41598-019-51285-8)
Supplement: Supplementary file 2 — supplementary tables [file 41598_2019_51285_MOESM2_ESM.pdf]

Metabolic Biomarkers In Midtrimester Maternal Plasma Can Accurately Predict Adverse Pregnancy Outcome in Patients with SLE

Seung Mi Lee, MD, PhD[1], Eun Mi Lee, Ms[2], Jin Kyun Park, MD, PhD [3], Hae Sun Jeon, MD[1], Sohee Oh, PhD[4], Subeen Hong, MD[1], Young Mi Jung, MD[1], Byoung Jae Kim, MD[1,5], Sun Min Kim, MD, PhD[1,5], Errol R. Norwitz, MD, PhD[6], Eun Bong Lee, MD, PhD [3], Souphaphone Louangsenlath, MD[7], Chan-Wook Park, MD, PhD [1], Jong Kwan Jun, MD, PhD [1], Joong Shin Park, MD, PhD[1*], Do Yup Lee, PhD[2*]

[1] Department of Obstetrics and Gynecology, Seoul National University College of Medicine, Seoul, Korea

[2] Department of Agricultural Biotechnology, Seoul National University, Seoul, Korea

[3] Division of Rheumatology, Department of Internal Medicine, Seoul National University College of Medicine, Seoul, Korea

[4] Department of Biostatistics, Seoul Metropolitan Government Seoul National University Boramae Medical Center, Seoul, Korea

[5] Department of Obstetrics and Gynecology, Seoul Metropolitan Government Seoul National University Boramae Medical Center, Seoul, Korea

[6] Department of Obstetrics and Gynecology, Tufts University School of Medicine, Boston, MA, U.S.A

[7] Department of Obstetrics and Gynecology, University of health science, Lao PDR

Supplementary table 1. Univariate analysis of metabolomics profiles between pregnant women with SLE and normal pregnancy outcome (group 1) and pregnant women with SLE with subsequent adverse pregnancy outcome (group 2)

Metabolites list	Fold change	P value[a]	Q value[b]
Lipid molecules			
ACar C6:0	2.28	0.044	0.057
ACar C10:2	1.82	0.033	0.057
ACar C11:1	1.88	0.036	0.029
ACar C14:0	2.13	0.036	0.037
ACar C16:0	1.46	0.022	0.037
ACar C16:4	1.95	0.014	0.037
ACar C22:6	1.72	0.024	0.111
FA C14:0	1.28	0.044	0.303
FA C16:1	2.84	0.003	0.037
FA C17:0	1.40	0.011	0.037
FA C18:1	2.67	0.013	0.029
FA C18:3	2.95	0.011	0.037
FA C20:4	2.42	0.033	0.037
FA C22:5	6.52	0.001	0.029
FA C22:6	3.63	0.001	0.037
FA C24:0	1.31	0.036	0.057
LysoPC C14:0	0.18	0.009	0.437

LysoPC C15:0	0.38	0.033	0.402
LysoPC C16:1	0.30	0.009	0.437
LysoPC C16:2	0.61	0.010	0.474
LysoPC C18:2	0.59	0.030	0.241
LysoPC C18:3	0.33	0.008	0.241
LysoPC C20:4	1.26	0.036	0.081
LysoPC C20:5	1.25	0.040	0.303
LysoPC C22:5	1.60	0.013	0.037
LysoPC C22:6	1.53	0.001	0.037
LysoPE C18:0	1.30	0.048	0.057
LysoPE C18:2	0.57	0.030	0.241
LysoPE C22:5	2.23	0.044	0.111
LysoPE C22:6	1.52	0.044	0.081
OxFA C18:0+1O(1Cyc)	2.16	0.027	0.057
OxFA C20:4+1O(1Cyc)	1.79	0.005	0.333
OxPC C34:3+1O	2.57	0.013	0.333
OxPC C36:4+1O(1Cyc)	1.54	0.014	0.242
OxPC C40:5+1O(1Cyc)	0.45	0.007	0.241
PC C24:6	2.35	0.016	0.057
PC C34:3	0.55	0.033	0.276
PC C35:5	0.47	0.002	0.276
PC C36:6	0.61	0.005	0.241
PC C38:5	0.56	0.024	0.241

PE C22:1	0.96	0.013	N/A
PE C24:0	0.99	0.022	N/A
PE C36:4	0.53	0.014	0.241
PE C38:5	0.56	0.030	0.241
PtdIns C34:1	0.64	0.044	0.402
SM C30:2	0.58	0.048	0.410
Primary metabolites			
2-deoxytetronic acid	1.75	0.011	0.037
Glyceric acid	0.73	0.036	0.276
Glycerol-alpha-phosphate	1.69	0.024	0.037
Lysine	0.70	0.003	0.241
Malic acid	1.38	0.013	0.029
Methionine	0.72	0.027	0.402
Proline	0.60	0.022	0.241
Tryptophan	0.65	0.001	0.000
Tyrosine	0.79	0.022	0.241

ACar, acylcarnitine; FA, free fatty acid; LysoPC, lysophosphatidylcholine; LysoPE, lysophosphatidylethanolamine; OxFA, oxidized fatty acid; OxPC, oxidized phosphatidylcholine; PC, phosphatidylcholine; PE, phosphatidylethanolamine; PtdIns, phosphatidylinositol; SM, sphingomyelin.

[a] P value is computed based on Mann-Whitney U test.

[b] Q value is calculated based on false discovery rate estimation.
